# A diagnostic scoring system for differentiating between benign and malignant cystic ovarian tumors, utilizing imaging features and biomarkers

**DOI:** 10.3389/fmed.2025.1720933

**Published:** 2026-01-12

**Authors:** Xiaozhong Zheng, Yunzhi Zhao, Anqi Wu, Jing Zhou, Yihua Wang, Mengyin Gu, Min Lin, Jianxia Xu

**Affiliations:** 1 Department of Radiology, The Second Affiliated Hospital of Zhejiang Chinese Medical University, Hangzhou, Zhejiang, China; 2 The First School of Clinical Medicine, School of Information and Engineering, Wenzhou Medical University, Wenzhou, China; 3 The Second Affiliated School of Zhejiang Chinese Medical University, Hangzhou, Zhejiang, China; 4 Department of Radiology, The Affiliated Hospital of Hangzhou Normal University, Hangzhou, Zhejiang, China

**Keywords:** benign cystic ovarian tumors, malignant cystic ovarian tumors, scoring system, imaging features, biomarkers

## Abstract

**Objective:**

Our study aims to establish and validate a diagnostic scoring system for distinguishing malignant cystic ovarian tumors (MCOTs) from benign cystic ovarian tumors (BCOTs).

**Methods:**

The study population was sourced from two independent hospitals. The subjects included 159 patients with 196 masses (137 in the training cohort and 59 in the validation cohort) who had undergone MRI or CT examinations with pathologically confirmed BCOT or MCOT. Four clinical characteristics, four biomarkers, and 16 imaging features were collected. Univariate analyses and multivariate logistic regression analyses were conducted to identify independent predictors for differentiating MCOTs from BCOTs. The independent predictors were weighted based on regression coefficients to construct a scoring system. The overall score distribution was categorized into three groups to illustrate the diagnostic probability of MCOTs.

**Results:**

The scoring system consisted of four independent predictive factors, including CA125, and three imaging features: texture, septum thickness, and enhancement degree. The area under the curve (AUC) for the scoring system was 0.956 (95% CI 0.926–0.986; *p* < 0.001), comparable to that of the primary predictive model at 0.971 (95% CI 0.949–0.993; *p* < 0.001). Utilizing 6.5 points as the cut-off value, a sensitivity of 86.6% and a specificity of 91.4% were achieved. The number of patients with MCOT in the three groups significantly increased with higher scores.

**Conclusion:**

The established scoring system is reliable and convenient for distinguishing between MCOTs and BCOTs by utilizing elevated CA125 levels, cystic-solid components, septum thickness≥4 mm, and a moderate or prominent degree of enhancement.

## 1 Introduction

Ovarian malignant tumors are prevalent diseases in the female reproductive system, characterized by high incidence and poor prognosis. Due to the ovaries’ anatomical position, early-stage patients often present no obvious symptoms. Most lesions have already metastasized to the pelvic cavity, and only conservative treatment can control the condition, yet the 5-year survival rate remains low. Therefore, improving early diagnosis rates for ovarian cancer patients is crucial to prolong survival time and enhance prognosis (1–4).

There are numerous histological types of ovarian tumors; the WHO classification of tumors 5th edition lists approximately 70 histological types (5). The large number of histological types means that it can be difficult to be familiar with the imaging findings of all types of ovarian tumors. A recent study noted that approximately 80% of ovarian masses contain the cystic component, a finding that highlights the need for in-depth investigation of tumors containing the cystic component (6).

Ultrasonography (US) is the first-line imaging modality for ovarian lesions and is a useful preoperative test for the characterization of noncomplex masses (7). Magnetic resonance imaging (MRI) and computed tomography (CT) can reveal morphologic characteristics such as papillary projections, nodularity, septum, and solid portions, but none of these criteria reliably distinguish between benign and malignant tumors (8–12).

Biomarkers, such as carbohydrate antigen 125 (CA125), carbohydrate antigen 19–9 (CA19-9), and carcinoembryonic antigen (CEA), all have potential value in early-stage ovarian cancer detection. CA125 is the most commonly used tumor biomarker for the diagnosis of ovarian tumors (13, 14). Currently, some studies focus solely on the differences in CA125 levels between benign and malignant tumors, neglecting comprehensive analysis of various tumor biomarkers, particularly when these markers are combined with imaging features (15, 16).

Based on this background, this study aims to develop and validate a diagnostic scoring system based on imaging features, clinical characteristics and biomarkers for distinguishing between benign cystic ovarian tumors (BCOTs) and malignant cystic ovarian tumors (MCOTs).

## 2 Materials and methods

### 2.1 Patients

**Figure 1:**
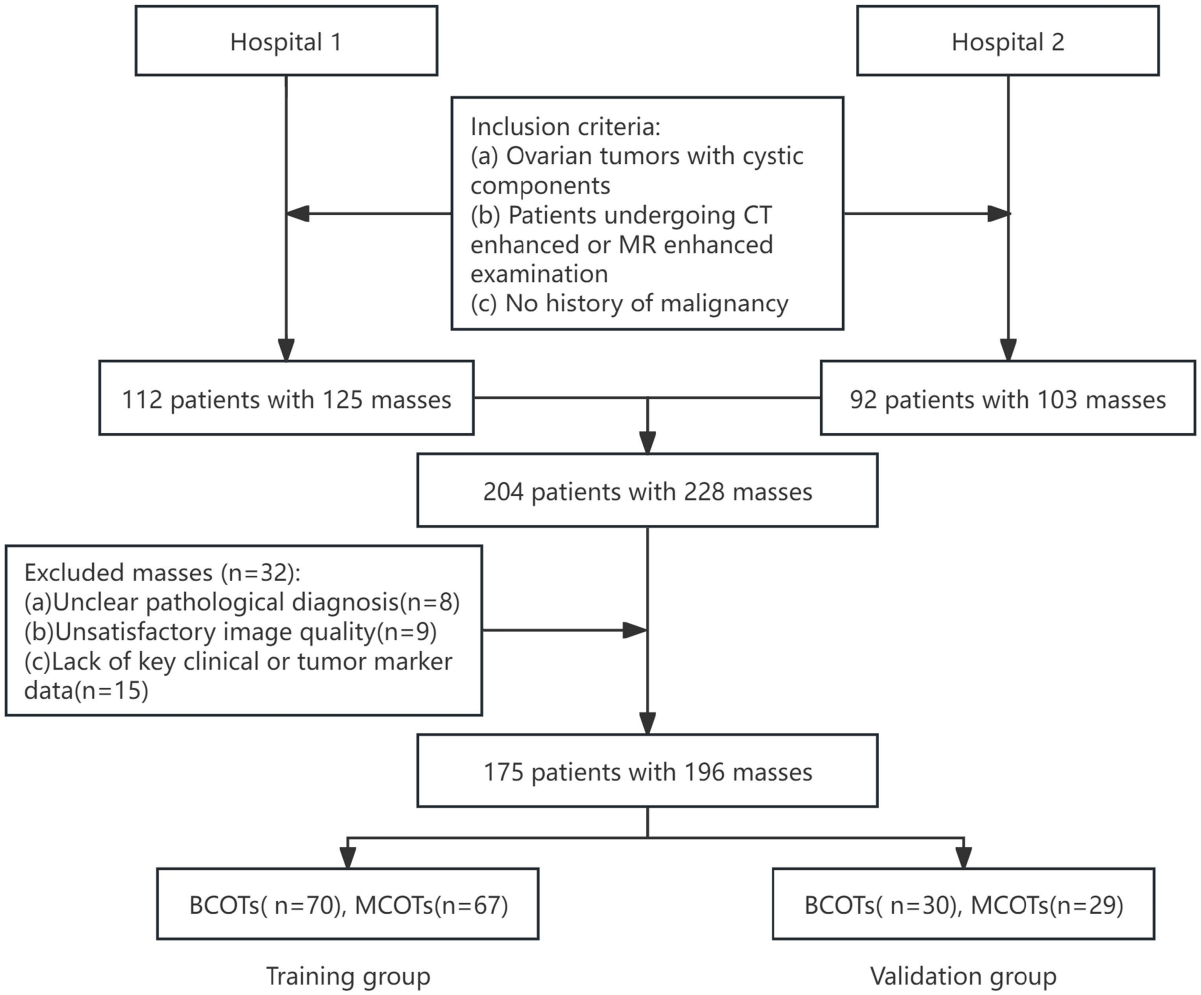
Flowchart of patient selection.

We retrospectively enrolled patients diagnosed with BCOT or MCOT by pathology at two hospitals between January 2018 and December 2023. Patients were randomly assigned to the training cohort and validation cohort at a 7:3 ratio. The study was conducted in accordance with the Declaration of Helsinki and was approved by the Ethics Committee (Number ID: 2025–070-01). The requirement for written informed consent was waived because of the observational and retrospective nature of this study. The following inclusion criteria were applied: (a) ovarian tumors with cystic components; (b) patients undergoing CT enhanced or MR enhanced examination (the use of MRI or CT is based on routine clinical practice and scanner availability at each facility, rather than on a prior assessment of the tumor’s nature); (c) no history of malignancy. The following patients were also excluded: (a) unclear pathological diagnosis; (b) unsatisfactory image quality; (c) lack of key clinical or tumor marker data (Figure 1).

### 2.2 Clinical data and biomarker collection

All patients were performed with required examinations. The clinical data and biomarkers included: age, BMI, alpha-fetoprotein (AFP) elevated (≥8.0 ng/mL), carcinoembryonic antigen (CEA) elevated (≥5.0 ng/mL), carbohydrate antigen 19–9 (CA19-9) elevated (≥37.0 U/mL), carbohydrate antigen 125 (CA125) elevated (≥35.0 U/mL), and carbohydrate antigen 15–3 (CA15-3) elevated (≥31.3 U/mL).

### 2.3 MRI and CT protocols

All patients underwent a pelvic MRI or CT examination. MR images were performed using a 1.5-T MRI system including Magnetom Avanto (Siemens Healthcare) or Signa HDxt (GE Healthcare). The MRI protocol included axial T1-weighted imaging (T1WI) including spin-echo with and without fat saturation (FS) (TR/TE, 420 ms/6.6 ms and 512 ms/7 ms, respectively), T2-weighted imaging (T2WI) turbo spin-echo with FS (TR/TE, 4000 ms/81 ms); and sagittal T2WI turbo spin-echo with FS (TR/TE, 3300 ms/73 ms). Contrast-enhanced T1WI with FS (TR/TE, 420 ms/6.6 ms) was performed in the axial, sagittal and coronal planes at 40–60 s (early phase), 90–110 s (middle phase), and 170–190 s (late phase) after the intravenous administration of 0.2 mmol/kg gadopentetate dimeglumine (Magnevist, Bayer Schering, Berlin, Germany) at a rate of 2–3 mL/s. The scanning parameters were as follows: 5 mm slice thickness, 1.5 mm gap, 256 × 256 matrix, 340 cm field of view, and four excitations. The area scanned extended from the inferior pubic symphysis to the renal hilum.

CT examinations were performed using Optima CT540 (GE Medical Systems). All examinations were performed after intravenous contrast (80–100 mL iohexol, 300 mg iodine/mL). The contrast agent was injected at the rate of 2.0–3.0 mL/s by using an automatic power injector. Patients were scanned on CT scanners with the following parameters: 100 mAs; 120 kV; helical pitch of 5 mm; thickness of 5 mm. Each patient underwent 3-phase contrast enhanced CT examinations of the pelvic cavity. The arterial phase was acquired with a delay time of 13–17 s after abdominal aorta attenuation reached 100 Hounsfield unit (Hu) using the tracking technique. Portal phase was performed 30 s after the arterial phase. Equilibrium phase scanning was performed 180 s after the portal phase.

### 2.4 Image analysis

All images were interpreted by two experienced abdominal radiologists (with 4 and 8 years of experience, respectively) independently and retrospectively. The radiologists were fully blinded to all clinical information (including age, BMI, menopausal status, etc.) and tumor biomarkers (including CA125, CA19-9, CA15-3, etc.), only knew the examination method (CT or MRI), and were equally unaware of the pathological results. In cases of initial disagreement, the two radiologists discussed findings to consensus. The following tumor imaging features were assessed: (a) maximum diameter of tumor; (b) maximum diameter of solid portion; (c) maximum diameter ratio of solid portion; (d) location; (e) texture; (f) shape; (g) margin; (h) cystic-solid interface; (i) number of cysts; (j) septum thickness; (k) ipsilateral ovary; (l) ascites; (m) lymph node; (n) enhancement degree. Texture definition: clearly divided into “cystic” and “cystic-solid”; cystic defined as the absence of any identifiable solid nodules or wall nodules/papillary protrusions within the lesion (Supplementary Figure 1); cystic-solid defined as the presence of continuous or discontinuous high-density/high-signal solid components within the lesion (Supplementary Figure 2). Cystic-solid interface definition: according to whether the boundary between solid and cystic components is clear (Supplementary Figures 3,4), pure cystic masses have no cystic-solid interface. Enhancement degree definition: relative to the normal myometrium at the same level, the enhancement degree is classified into “no or mild” (no enhancement or enhancement lower than the myometrium) and “moderate or prominent” (enhancement similar to or higher than the myometrium) (Supplementary Figures 5, 6). Septum thickness definition: the thickest septal layer is selected, and its thickness is measured along the direction perpendicular to the septal long axis on the clearest layer of the enhanced scan (Supplementary Figure 7).

### 2.5 Statistical analysis

Continuous variables were presented as either the mean±standard deviation (SD) in cases of normal distribution or the median and interquartile range (IQR) for cases with nonnormally distributed data. Categorical data were recorded as frequency (percentage). Student’s t-test was used for continuous variables with normal distribution, while Mann–Whitney U test was applied for data with non-normal distribution and the chi-square or Fisher’s exact test was used for categorical variables. Interobserver agreements for MRI or CT features were evaluated using the kappa statistic (k) for qualitative variables and the intra-class correlation coefficient (ICC) for quantitative variables. Given the large number of imaging variables in this study and their partial correlations, we first performed a preliminary screening for multicollinearity using the variance inflation factor (VIF). Ridge regression was then employed to reduce coefficients and improve model stability, followed by multivariate logistic regression to identify independent predictors for MCOTs and BCOTs (17). To establish an easy to calculate scoring system, the regression coefficients are rounded to the nearest integer and converted into weighted scores (18). For each patient, the scores corresponding to the relevant variables were summed to produce an overall score. Calibration was assessed using the Hosmer-Lemeshow goodness-of-fit test, and *p* > 0.05 indicated insignificant deviance from the theoretical perfect calibration in the training and validation groups. The discrimination performance of the model was assessed using the area under the receiver operating curve (AUC), and the optimum cutoff point was chosen for optimal sensitivity and specificity. A comparison between the AUCs of different models was performed using the DeLong nonparametric method. To evaluate the robustness of the proposed scoring system across different imaging modalities, a prespecified sensitivity analysis was performed by stratifying patients into MRI-only and CT-only subgroups according to the imaging modality used for diagnosis. Within each subgroup, the diagnostic performance of the scoring system was assessed using receiver operating characteristic (ROC) curve analysis, and the area under the curve (AUC) was calculated. *p* values <0.05 were considered to indicate a significant difference. Except for ROC comparison using MedCalc Software version 22.0, all data were analyzed using SPSS version 23.0.

## 3 Results

### 3.1 Clinical characteristics and biomarkers in patients

The subjects consisted of 159 patients with 196 masses. A total of 122 patients had one mass, and 37 patients had two masses. Among them, 100 were benign cystic ovarian tumors (BCOTs) and 96 were malignant cystic tumors (MCOTs). The histological subtypes of BCOTs and MCOTs are summarized in Table 1. The 196 masses included in this study covered 13 histological subtypes, among which the benign tumors were mainly serous cystadenoma (67/100) and mature cystic teratoma (20/100), and the malignant tumors were mainly serous cystadenocarcinoma (58/96) and mucinous cystadenocarcinoma (19/96). The heterogeneity of subtypes was in line with the clinical characteristics of the disease. Patients with BCOT and MCOT were randomly assigned 7:3 into training group (BCOTs *n* = 70, MCOTs *n* = 67) and validation group (BCOTs *n* = 30, MCOTs *n* = 29). In the training cohort, the levels of CA125 and CA15-3 in the MCOT group were higher than those in the BCOT group (*p* < 0.05). There were no significant differences in age, BMI, menopause, or the levels of AFP, CEA, and CA19-9 between the BCOT and MCOT groups (Table 2).

**Table 1:** Distribution of histological subtypes of cystic ovarian tumor.

Type of cystic ovarian tumors	*N* = 196
Benign cystic ovarian tumors	100
Serous cystadenoma	67
Mucinous cystadenoma	6
Brenner tumor	1
Clear cell tumor	2
Struma ovarii	1
Thecoma-fibroma tumor	3
Mature cystic teratoma	20
Malignant cystic ovarian tumors	96
Serous cystadenocarcinoma	58
Mucinous cystadenocarcinoma	19
Clear cell carcinoma	10
Granulosa cell tumor	2
Yolk sac tumor	5
Endometrioid carcinoma	2

**Table 2:** Comparison of patients in clinical characteristics and biomarkers in the training cohort.

Variables	BCOT (*n* = 70)	MCOT (*n* = 67)	*p* value
Age (years)	54.67 ± 17.68	55.81 ± 14.59	0.683
BMI	23.58 ± 4.01	24.06 ± 3.23	0.442
Menopause	40 (57.1%)	32 (48.5%)	0.312
AFP elevated	3 (4.3%)	6 (9.0%)	0.270
CEA elevated	8 (11.4%)	6 (9.0%)	0.633
CA125 elevated	12 (17.1%)	49 (73.1%)	**<0.001**
CA15-3 elevated	7 (10.0%)	22 (32.8%)	**0.001**
CA19-9 elevated	15 (21.4%)	12 (17.9%)	0.605

Bold values indicate statistical significance, with *P* < 0.05.

### 3.2 Imaging features

A univariate analysis of imaging features was conducted to identify the most pertinent predictors for distinguishing patients with MCOT from those with BCOT in the training cohort. Eleven imaging variables, including the maximum diameter of the solid portion (*p* < 0.001), the maximum diameter ratio of the solid portion (*p* < 0.001), location (*p* = 0.003), texture (*p* < 0.001), shape (*p* < 0.001), margin (*p* < 0.001), cystic-solid interface (*p* < 0.001), septum thickness (*p* < 0.001), enhancement degree (*p* < 0.001), ascites (*p* < 0.001), and lymph node (*p* < 0.001), were found to be significantly different between BCOT and MCOT patients (Table 3). The 95% confidence intervals (CIs) for these variables are provided in the Supplementary Table 1. Interobserver agreement for all imaging variables was good to excellent (kappa = 0.811–1.000, ICC = 0.854–0.937; Table 4). Among them, the kappa value of septum thickness was 0.811, the kappa value of enhancement degree was 0.870, the kappa value of texture was 0.971, and the kappa value of cystic-solid interface was 0.982, indicating that the image features on which the scoring system depends have good repeatability.

**Table 3:** Comparison of the imaging variables between BCOTs and MCOTs in the training cohort.

Variables	BCOT (*n* = 70)	MCOT (*n* = 67)	*p* value
Maximum diameter of tumor (mm)	8.23 ± 5.62	9.41 ± 5.65	0.223
Maximum diameter of solid portion (mm)	1.32 ± 1.08	3.96 ± 3.02	**<0.001**
Maximum diameter ratio of solid portion	0.12 ± 0.08	0.46 ± 0.24	**<0.001**
Location			
Bilateral/unilateral	9/61	23/44	**0.003**
Texture			
Cystic/cystic-solid	46/24	1/66	**<0.001**
Shape			
Round or oval/irregular	57/13	16/51	**<0.001**
Margin			
Smooth/unsmooth	67/3	46/21	**<0.001**
Cystic-solid interface			
Without/clear/unclear	47/18/5	1/36/30	**<0.001**
Number of cysts			
<10/10–20/>20	59/6/5	49/6/12	0.176
Septum thickness			
<4 mm/≥4 mm	60/10	30/37	**<0.001**
Hemorrhage			
Absence/presence	62/8	59/8	0.926
Enhancement degree			
No or mild/moderate or prominent	44/26	12/55	**<0.001**
Ipsilateral ovary			
Absence/presence	54/16	60/7	0.052
Ascites			
No or physiological/moderate/massive	50/20/0	35/12/20	**<0.001**
Lymph node			
<1 cm/≥1 cm	70/0	51/16	**<0.001**

Bold values indicate statistical significance, with *P* < 0.05.

**Table 4:** Interobserver agreement of imaging variables.

Variables	*k* value	ICC
Maximum diameter of tumor	–	0.937
Maximum diameter of solid portion	–	0.854
Maximum diameter ratio of solid portion	–	0.916
Location	1.000	–
Texture	0.971	–
Shape	0.936	–
Margin	0.960	–
Cystic-solid interface	0.982	–
Number of cysts	0.948	–
Septum thickness	0.811	–
Hemorrhage	0.869	–
Enhancement degree	0.870	–
Ipsilateral ovary	0.948	–
Ascites	0.956	–
Lymph node	0.845	–

### 3.3 Establishment of the primary predictive model

**Figure 2:**
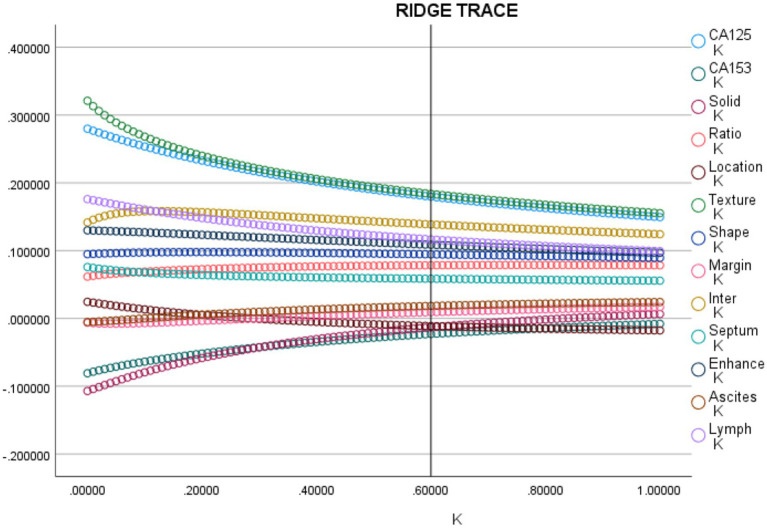
The ridge curve of the relevant predictors for distinguishing MCOT from BCOT.

Variables identified as significantly different in the univariate analysis were included in the ridge regression analysis to minimize multicollinearity. As presented in the ridge trace curve (Figure 2), when the K value was 0.6, the ridge trace presented with the standardized coefficients of variables was stable, and the model was significant (*p* < 0.001). At this point, eight features showed significant differences between BCOT and MCOT patients (Table 5). For further verification, multivariate logistic regression was performed to demonstrate four independent factors: CA125 (*p* < 0.001), texture (*p* = 0.008), septum thickness (*p* = 0.016), enhancement degree (*p* = 0.005; Table 6). These four factors were adopted to develop the scoring model. The Hosmer-Lemeshow goodness-of-fit test indicated good calibration for this primary model (*p* = 0.759), and the AUC for the primary predictive model was 0.971 (95% CI: 0.949–0.993; *p* < 0.001).

**Table 5:** Ridge regression results of clinical and imaging features (*k* = 0.60).

Variables	*B*	SE	Beta	B/SE(B)	*p* value
CA125	0.182	0.034	0.181	5.344	**<0.001**
CA15-3	−0.029	0.041	−0.024	−0.724	0.469
Maximum diameter of solid portion	−0.002	0.004	−0.015	−0.468	0.640
Maximum diameter ratio of solid portion	0.137	0.052	0.078	2.620	**0.009**
Location	−0.011	0.039	−0.010	−0.300	0.764
Texture	0.194	0.030	0.184	6.274	**<0.001**
Shape	0.091	0.033	0.091	2.761	**0.006**
Margin	0.005	0.043	0.004	0.131	0.895
Cystic-solid interface	0.087	0.018	0.135	4.839	**<0.001**
Septum thickness	0.044	0.017	0.084	2.510	**0.013**
Enhancement degree	0.109	0.034	0.107	3.142	**0.002**
Ascites	0.013	0.022	0.019	0.582	0.561
Lymph node	0.080	0.024	0.111	3.243	**0.001**
Constant	−0.281	0.109	0.000	−2.582	0.010

Bold values indicate statistical significance, with *P* < 0.05.

**Table 6:** Multivariate regression analysis for features and the weighted score of independent predictors.

Variables	*B*	*p* value	OR	95% CI for OR		VIF	Weightd Score
				Lower	Upper		
CA125 (elevated)	2.858	**<0.001**	17.432	3.965	76.636	1.22	3
Texture (cystic-solid components)	5.242	**0.008**	189.024	3.956	932.069	1.27	5
Septum thickness (≥4 mm)	0.887	**0.016**	2.429	1.015	5.814	1.19	1
Enhancement degree (moderate or prominent enhancement degree)	1.302	**0.005**	3.677	0.0875	15.453	1.23	1

Bold values indicate statistical significance, with *P* < 0.05.

### 3.4 Establishment of the scoring system

**Figure 3:**
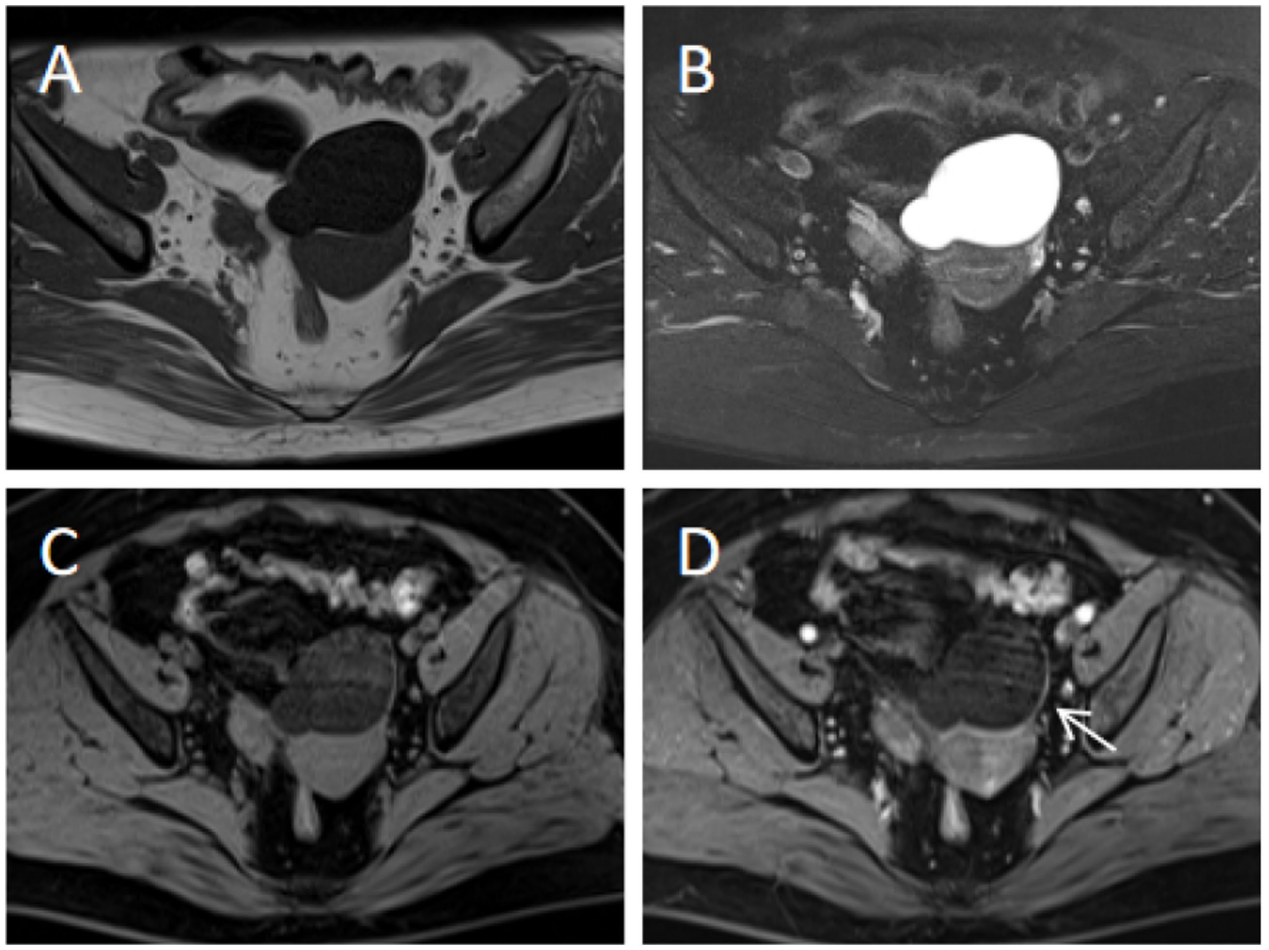
60-year-old patient with left ovarian serous cystadenoma (benign). **(A,B)** Axial T1WI and T2WI-FS show a pure cystic mass in the pelvis, with homogeneous signal intensity and no solid components. **(C,D)** Axial contrast-enhanced T1WI-FS (middle phase) demonstrates mild enhancement of the cyst wall (arrow) and no definite septum. Scoring rationale: CA125 level was normal (18.5 U/mL, 0 points), texture was cystic (0 points), septum thickness <4 mm (0 points), enhancement degree was mild (0 points). Therefore, a score of 0 was assigned for this patient.

**Figure 4:**
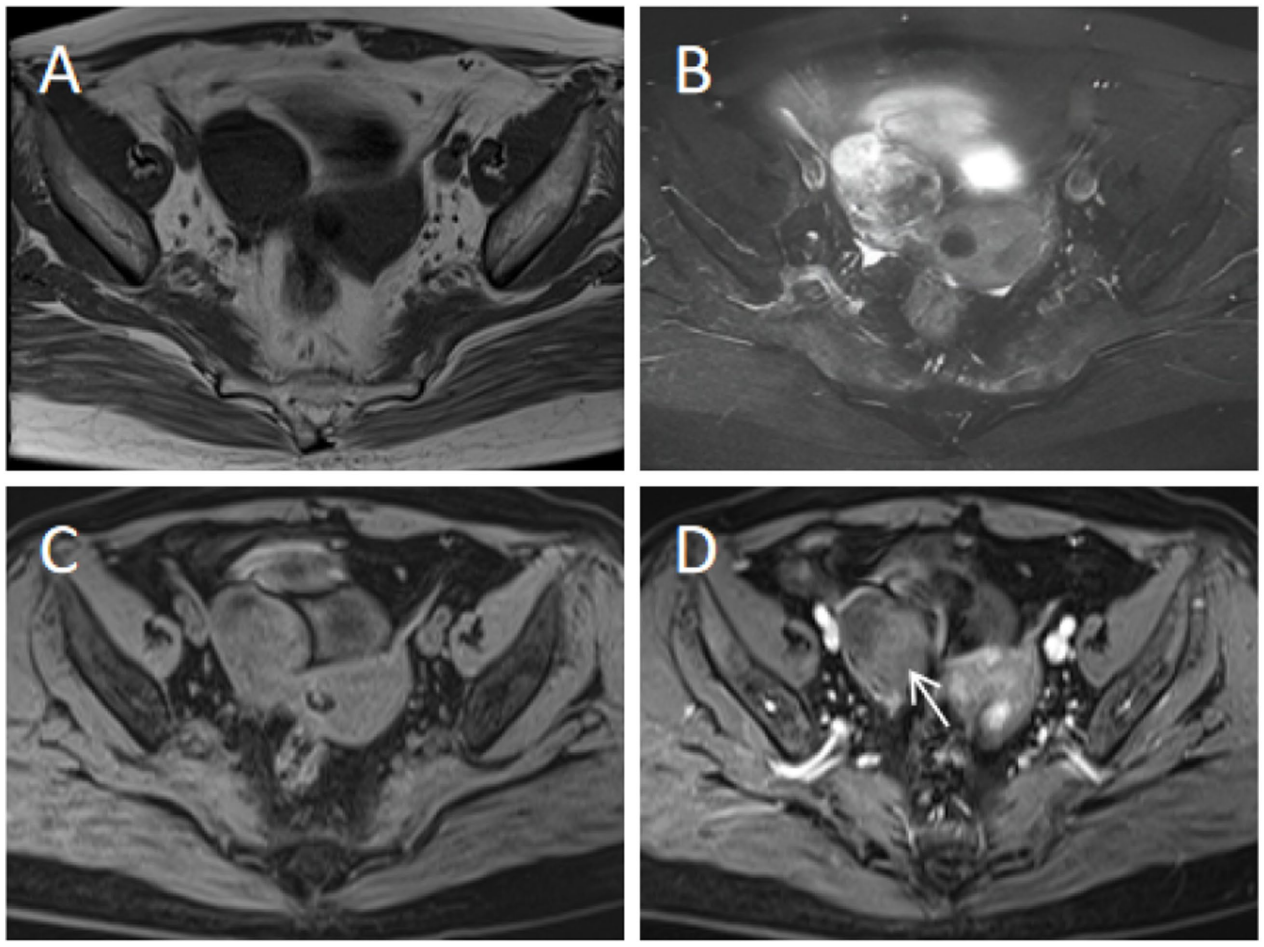
A 61-year-old patient with right ovarian thecoma-fibroma tumor (benign). **(A,B)** Axial T1WI and T2WI-FS reveal a cystic-solid mass in the right ovary, with a focal cystic component and a large solid component. **(C,D)** Axial contrast-enhanced T1WI-FS (middle phase) shows moderate enhancement of the solid component (arrow), with no thickened septa. Scoring rationale: CA125 level was normal (21.7 U/mL, 0 points), texture was cystic-solid (5 points), septum thickness <4 mm (0 points), enhancement degree was moderate (1 point). Therefore, a score of 6 was assigned for this patient.

**Figure 5:**
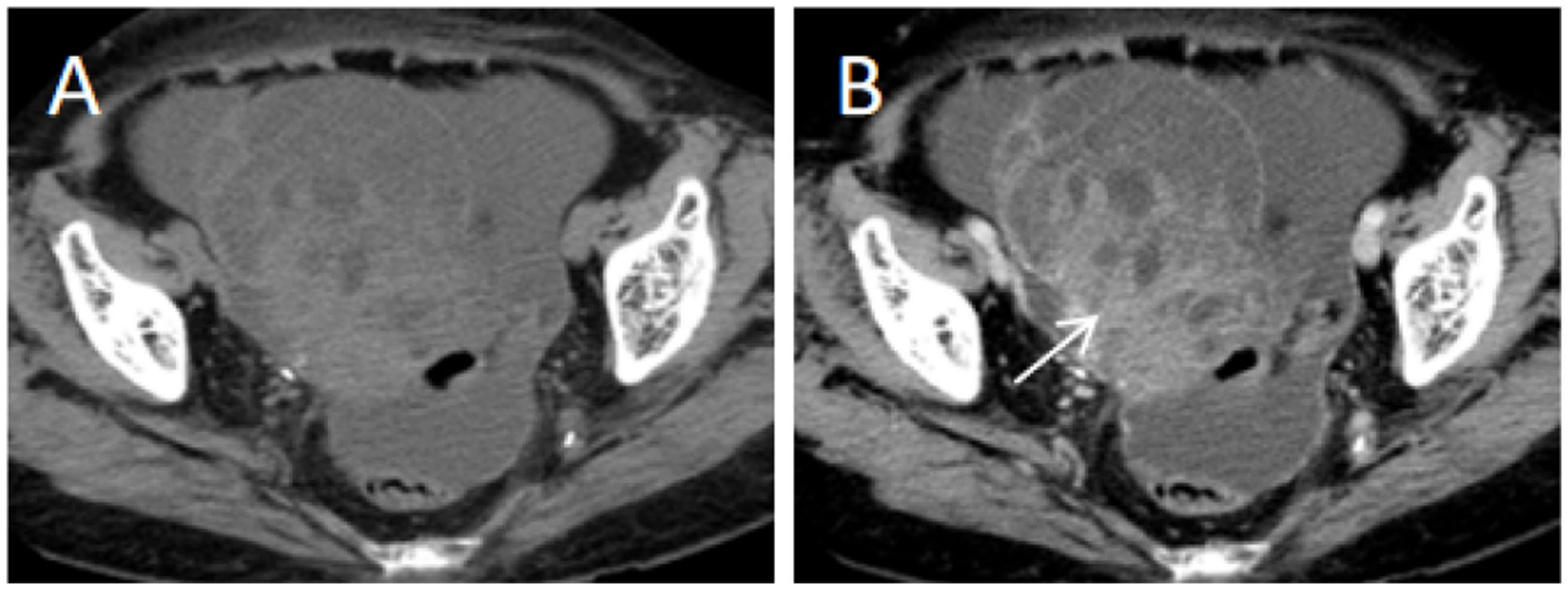
91-year-old patient with right ovarian mucinous adenocarcinoma (malignant). **(A)** Axial unenhanced CT image shows a cystic-solid mass in the right ovary, with multiple thickened septa (thickness ≥4 mm). **(B)** Axial contrast-enhanced CT image (portal venous phase) demonstrates prominent enhancement of the solid components (arrow). Scoring rationale: CA125 level was elevated (289.5 U/mL, 3 points), texture was cystic-solid (5 points), septum thickness ≥4 mm (1 point), enhancement degree was prominent (1 point). Therefore, a score of 10 was assigned for this patient.

**Figure 6:**
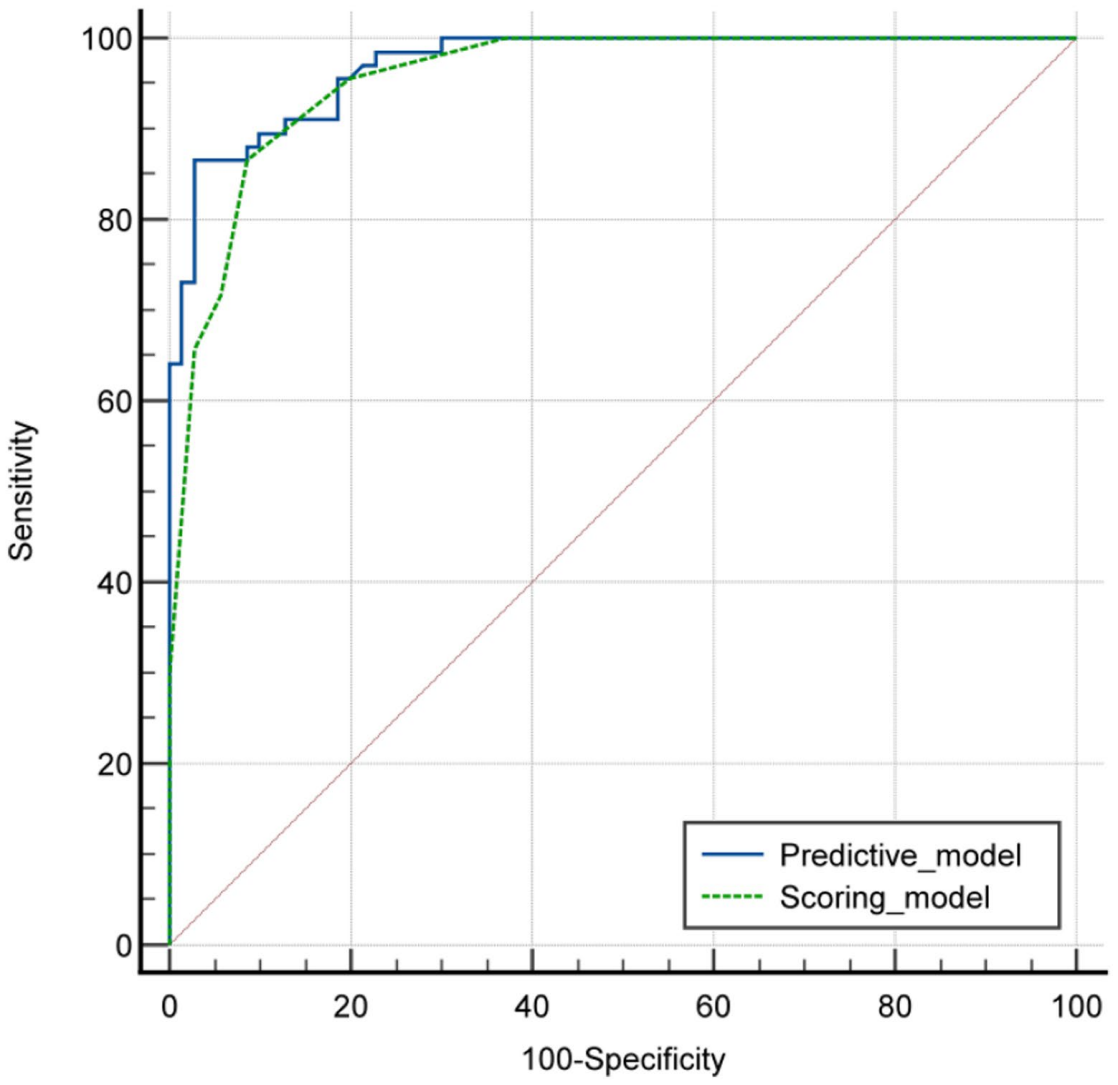
ROC curves of the predictive model and the score model.

To provide a quantitative tool for predicting risk classification, a predictive score model based on multivariate analysis within the training cohort was proposed. Weighted scoring was performed based on the regression coefficients of each variable, which showed statistical significance in multivariate analysis (Table 6). CA125 elevated was assigned 3 points; cystic-solid components was assigned 5 points; septum thickness ≥4 mm was assigned 1 point; moderate or prominent enhancement degree was assigned 1 point. For each patient, the scores corresponding to the predictors were summed to produce an overall score ranging from 0 to 10 points (Figures 3–5). The higher the score, the more likely the lesion was to be MCOT. The Hosmer-Lemeshow goodness-of-fit test indicated good calibration of this scoring model (*p* = 0.744). The AUC of this distinguishing scoring system, measured by receiver operating characteristic (ROC) curve analysis, was 0.956 (95% CI 0.926–0.986, *p* < 0.001). At a cutoff score of 6.5 points, the model’s performance showed a sensitivity of 86.6% and a specificity of 91.4%. The comparison of ROC curves indicated no significant difference between the primary predictive model and the score model (*p* = 0.134), suggesting that the score model effectively utilized the predictive value of the primary model (Figure 6).

To facilitate the practical application of this scoring system, we have divided it into three score ranges: 0–3 points, 4–7 points, and 8–10 points. Within these scoring ranges, the probability of patients with MCOT was 0% in the first range (0 to 3 points), 44.19% in the second range (4–7 points), and 92.31% in the last range (8–10 points; Table 7).

**Table 7:** Diagnostic probability of MCOT in different score ranges in the training and validation cohorts.

Score range	Number of MCOT		Total number		Diagnostic probability of MCOT	
	Training cohort	Validation cohort	Training cohort	Validation cohort	Training cohort	Validation cohort
0–3 points	0	1	42	13	0%	7.6%
4-7 points	19	8	43	22	44.19%	36.36%
8–10 points	48	20	52	24	92.31%	83.33%

### 3.5 Validation of the established scoring system

**Figure 7:**
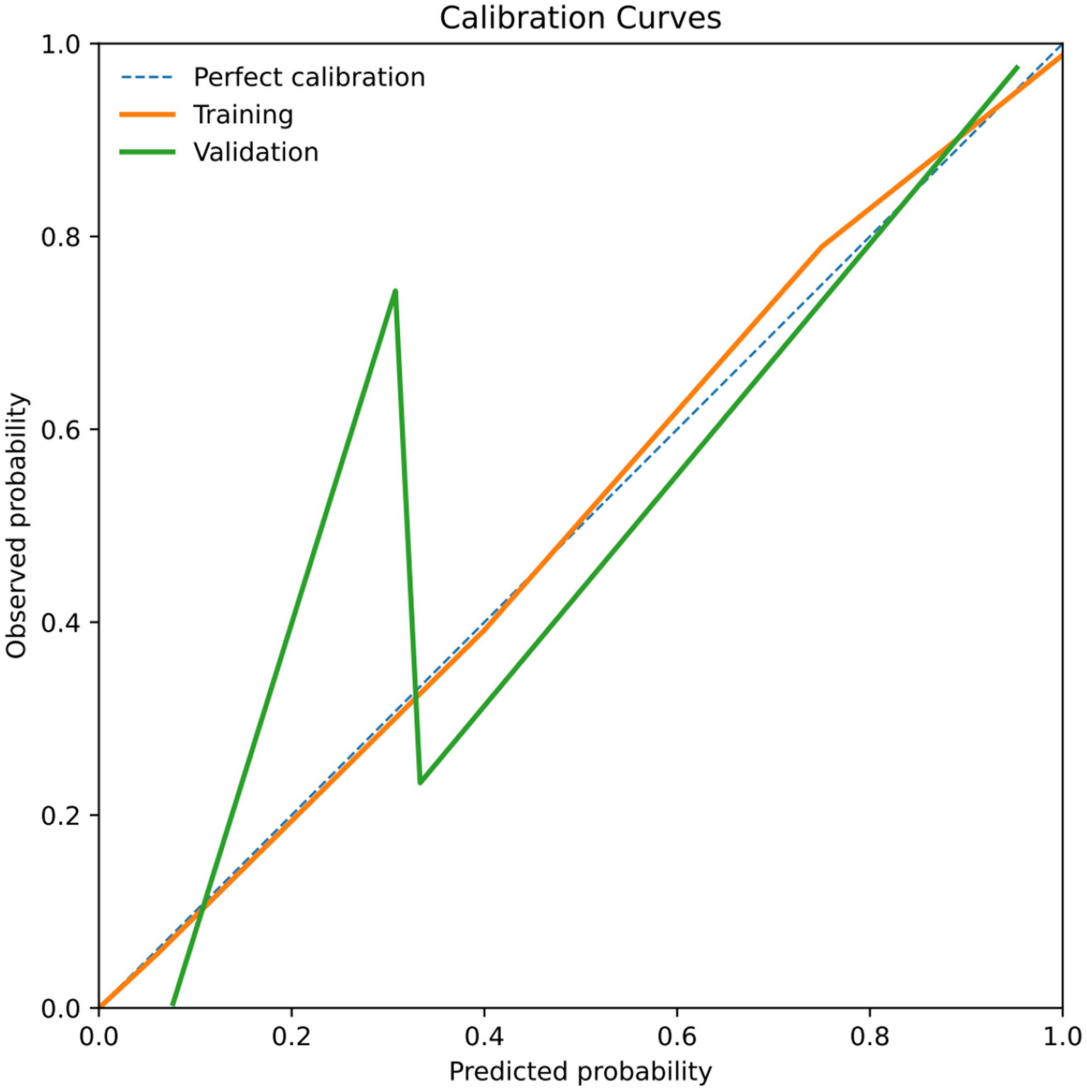
Calibration curves of the training cohort and validation cohort.

**Figure 8:**
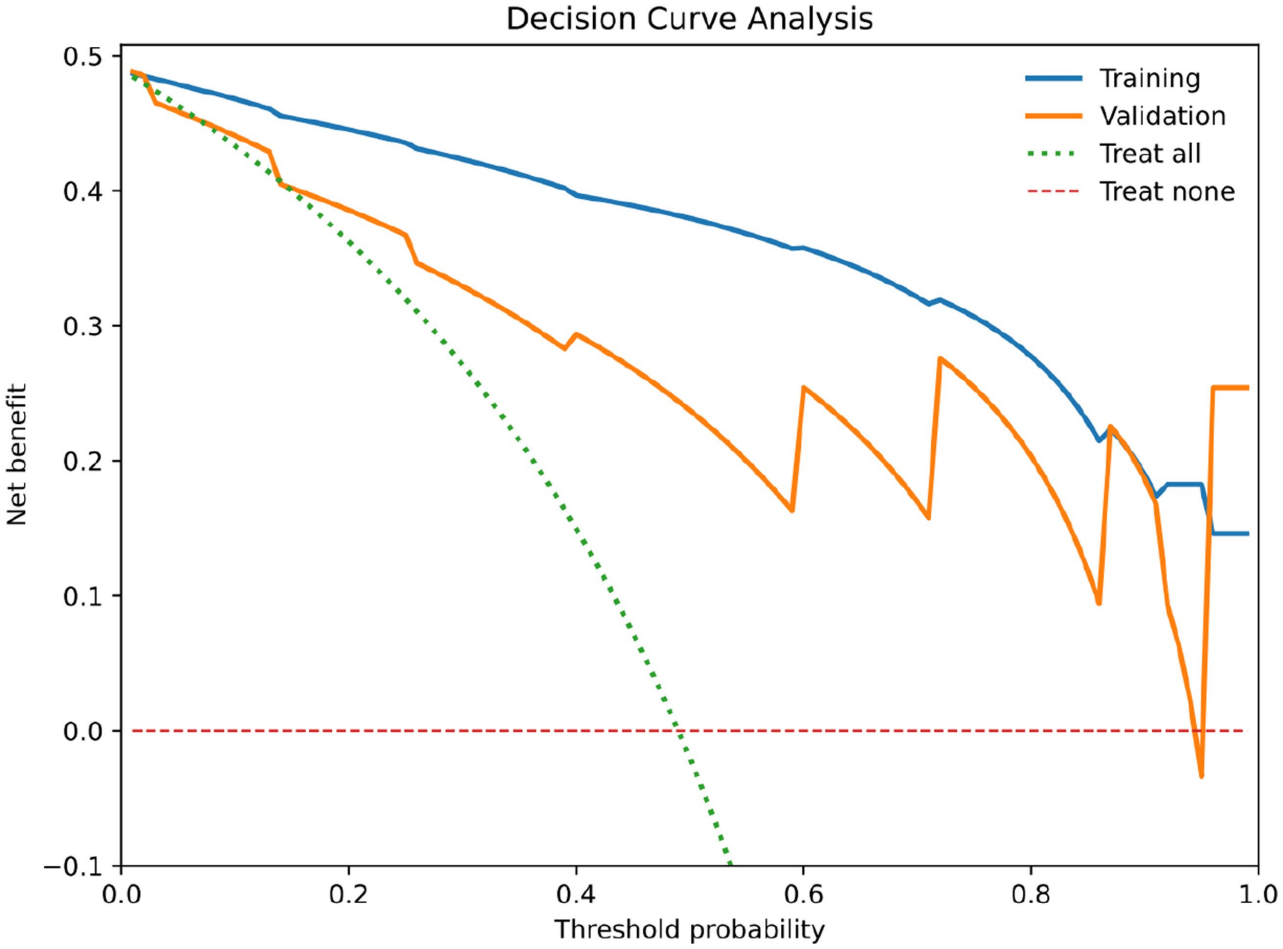
Decision curve analysis of the training cohort and validation cohort.

The validation of the scoring system yielded satisfactory results. The Hosmer-Lemeshow goodness-of-fit test indicated good calibration (*p* = 0.551). The AUC of the scoring system was 0.887 (95% CI 0.804–0.970, *p* < 0.001) in the validation cohort. At a cutoff score of 6.5 points, the model exhibited a sensitivity of 82.8% and a specificity of 80.7%, figures similar to those of the training cohort. In the validation cohort, the proportion of patients with MCOT increased with rising scores (Table 7). Calibration curves for both training and validation cohorts shown in Figure 7, demonstrating good agreement between predicted and observed probabilities. Decision-curve analysis (DCA) has also been included to assess clinical utility (Figure 8). In the training and validation cohorts, DCA analysis showed that compared to the default strategy (treating all patients or not treating any patients), this scoring model consistently provided higher net clinical benefits at most threshold probabilities. These results suggest that this detection combination may provide practical clinical advantages by reducing unnecessary invasive procedures and lowering the risk of diagnostic errors. We also applied the scoring system in clinical use (Supplementary Table 2, Supplementary Table 3).

### 3.6 Subgroup analysis by imaging modality

The ovarian masses were divided into subgroups according to imaging modalities, and finally divided into MRI-only subgroup (141 masses, 98 in training cohort and 43 in validation cohort) and CT-only subgroup (55 masses, 39 in training cohort and 16 in validation cohort). The baseline characteristics and diagnostic performance of the subgroups are detailed in Supplementary Table 4. Statistical analysis showed no significant difference in diagnostic performance between the two subgroups (AUC 0.948 vs. 0.923, *p* = 0.502). These results indicate that the discriminatory ability of the scoring system was comparable between CT and MRI, supporting its robustness across routine contrast-enhanced imaging modalities.

## 4 Discussion

This study established and validated a simple and practical preoperative scoring system to differentiate MCOTs from BCOTs. The final model integrated one serum biomarker (CA125) and three imaging features (texture, septum thickness, and enhancement degree) to form a scoring system with a total score ranging from 0 to 10. A higher score indicates a greater possibility of MCOT. This multimodal approach combines biochemical and morphological information, aiming to provide a reproducible and easily applicable tool for clinical practice, serving as a useful complement to existing structured imaging reporting systems (17–20).

Ultrasonography remains the first-line modality for the evaluation of adnexal masses; however, approximately 20% of lesions remain indeterminate following ultrasound assessment (21). Structured reporting systems, such as Gynaecologic Imaging Reporting and Data System (GI-RADS), and Ovarian-Adnexal Reporting and Data System (O-RADS), have improved communication between radiologists and clinicians (22, 23), but discrepancies in definitions, classification criteria, and quantitative thresholds limit their standardization and widespread adoption (24, 25). Our scoring system focuses on a limited number of imaging parameters. While CT and MRI inherently differ in soft tissue contrast, the imaging features selected for this scoring system, such as texture, septum thickness, and enhancement degree, are easily identifiable in routine clinical practice. The model demonstrates excellent consistency between two radiologists (kappa 0.811–1.000). Subgroup analysis further confirms the scoring system’s robustness across different imaging modalities. When ultrasonographic results are inconclusive, these variables can be objectively evaluated using CT or MRI, thereby addressing an existing diagnostic gap.

Our findings demonstrated significant morphological and quantitative differences between BCOTs and MCOTs. BCOTs typically appear as round or oval lesions with smooth margins, well-defined cystic-solid interfaces, and little or no ascites. In contrast, MCOTs often present as irregular or lobulated masses with thickened septum, indistinct interfaces, moderate to marked enhancement, and frequent ascites or bilateral involvement. Previous studies have also reported that internal complexity and strong enhancement are key imaging features suggesting malignant transformation in cystic ovarian tumors, consistent with our results (26, 27). These imaging characteristics correlate closely with histopathological features of MCOTs, where immature neovascularization within solid components results in more complex internal structures and conspicuous enhancement. In terms of quantitative parameters, the proportion of solid components in MCOTs was significantly higher than in BCOTs, whereas overall tumor size showed no statistical difference. This finding contrasts with earlier studies reporting larger volumes for malignant mucinous cystadenomas (27), likely due to our broader inclusion of diverse histological subtypes. Furthermore, as this study primarily conducted univariate analyses, the observed associations should be interpreted as potential correlations rather than independent predictors. Future statistical validation is required to determine whether these features serve as independent risk factors distinguishing MCOTs from BCOTs.

To establish a practical and reproducible predictive system, multivariate regression analysis was performed to obtain the relevant predictors that were significantly different between MCOTs and BCOTs. A total of four features were finally selected to build the scoring system, including three imaging features of texture, septum thickness, and enhancement degree, and one biomarker of CA125. The incidence of solid components was substantially higher in MCOTs, while most BCOTs were purely cystic. Prior research has similarly noted that benign epithelial ovarian tumors usually lack solid components, borderline lesions have limited solid parts, and malignant tumors are predominantly or entirely solid (26). In our study, the septum thickness of MCOTs was significantly greater than that of BCOTs, and ROC analysis determined 4 mm as the optimal diagnostic threshold, consistent with the 2–4 mm range reported in prior literature (16). This supports the reproducibility and cross-center applicability of this metric. Moreover, the solid components of MCOTs exhibited both greater proportion and stronger enhancement. Tumor enhancement reflects vascularity and neovascular permeability: malignant tumors with dense, permeable vessels demonstrate greater contrast retention and thus more pronounced enhancement on CT or MRI (28–30), aligning with our findings.

Regarding biochemical indices, multivariate logistic regression confirmed that elevated serum CA125 is an independent predictor of MCOT. Although CA125 remains the most widely used biomarker for malignant ovarian tumors screening and monitoring (13, 14), its specificity is limited, as levels may rise during menstruation, pregnancy, endometriosis, or peritonitis (31, 32). Therefore, reliance on CA125 alone risks false-positive diagnoses. In this study, CA125 was not used as a standalone predictor but as part of an integrated imaging-biomarker scoring system. The combination of CA125 with objective imaging features substantially improves specificity compared with CA125 alone. Menopausal status was not included in the final multivariate model due to limited incremental predictive value in univariate analysis; however, its potential confounding role is now discussed as a limitation and a direction for future research. Additionally, univariate analysis revealed that elevated CA15-3 occurred more frequently in MCOTs than in BCOTs (*p* = 0.001), consistent with prior reports linking elevated CA15-3 to advanced ovarian cancer (33). However, no significant differences were observed for CA19-9 or CEA. It is noteworthy that recent studies suggest that KRAS/BRAF mutations play critical roles in the subtyping and prognosis of ovarian tumors, pointing toward future directions for integrating radiomics with molecular biomarkers (34).

However, it is noteworthy that the scoring system still exhibits false-negative cases, where assessments tend to favor BCOT but correspond to MCOT in reality. These primarily involve early-stage or low-grade malignancies, such as early-stage serous cystic carcinoma and early-stage mucinous cystadenocarcinoma. These lesions are predominantly multilocular cystic with minimal solid components, and CA125 levels remain unremarkably elevated. Therefore, for patients with highly suspected malignancy but low scores, a comprehensive clinical evaluation and follow-up assessment should be conducted rather than relying solely on a single scoring tool.

This study has several limitations. First, this is a retrospective, single-country, multi-institutional study design, with inevitable histological selection bias, and no separate assessment of borderline ovarian tumors. Secondly, the heterogeneous imaging modalities, including contrast-enhanced CT and MRI, may still exhibit potential measurement heterogeneity despite our selection of image features that can be stably observed in both modalities. Third, no external multicenter validation has been performed: the model has been trained and validated only in data from two independent hospitals, and future work will need to validate the model’s generalizability across different countries/regions and more centers. Fourth, the potential overfitting caused by the small size of the validation cohort. Fifth, no comparison was made with existing standard systems (O-RADS MRI, IOTA Simplified Rules). Sixth, CA125-based prediction may be misleading in premenopausal women. Future studies should explore “menopausal status-based stratified thresholds” in larger cohorts to reduce false-positive rates. Seventh, this study did not perform stratified modeling for different histological subtypes. The current scoring system is primarily a “clinically-oriented comprehensive tool” that may exhibit variations across subtypes. This represents a critical direction for future validation, requiring larger sample sizes and specific subtype cohorts.

## 5 Conclusion

In summary, this study proposed and externally validated a reliable and convenient system. By combining CA125 elevated with three reproducible imaging features (cystic-solid components, septum thickness ≥4 mm and moderate or prominent enhancement degree), this system can effectively distinguish between MCOT and BCOT preoperatively. The model balances diagnostic accuracy with clinical practicality and can be readily incorporated into multidisciplinary decision-making processes involving radiologists and gynecologic oncologists to support surgical planning and patient counseling.

## Data Availability

The original contributions presented in the study are included in the article/Supplementary material, further inquiries can be directed to the corresponding author/s.
