# Supplementary material for: A diagnostic scoring system for differentiating between benign and malignant cystic ovarian tumors, utilizing imaging features and biomarkers
Source: Front Med (Lausanne). 2026 Jan 12;12:1720933. doi: 10.3389/fmed.2025.1720933 (PMC12832662; doi:10.3389/fmed.2025.1720933)
Supplement: Supplementary file 1 [file Supplementary_file_1.docx]

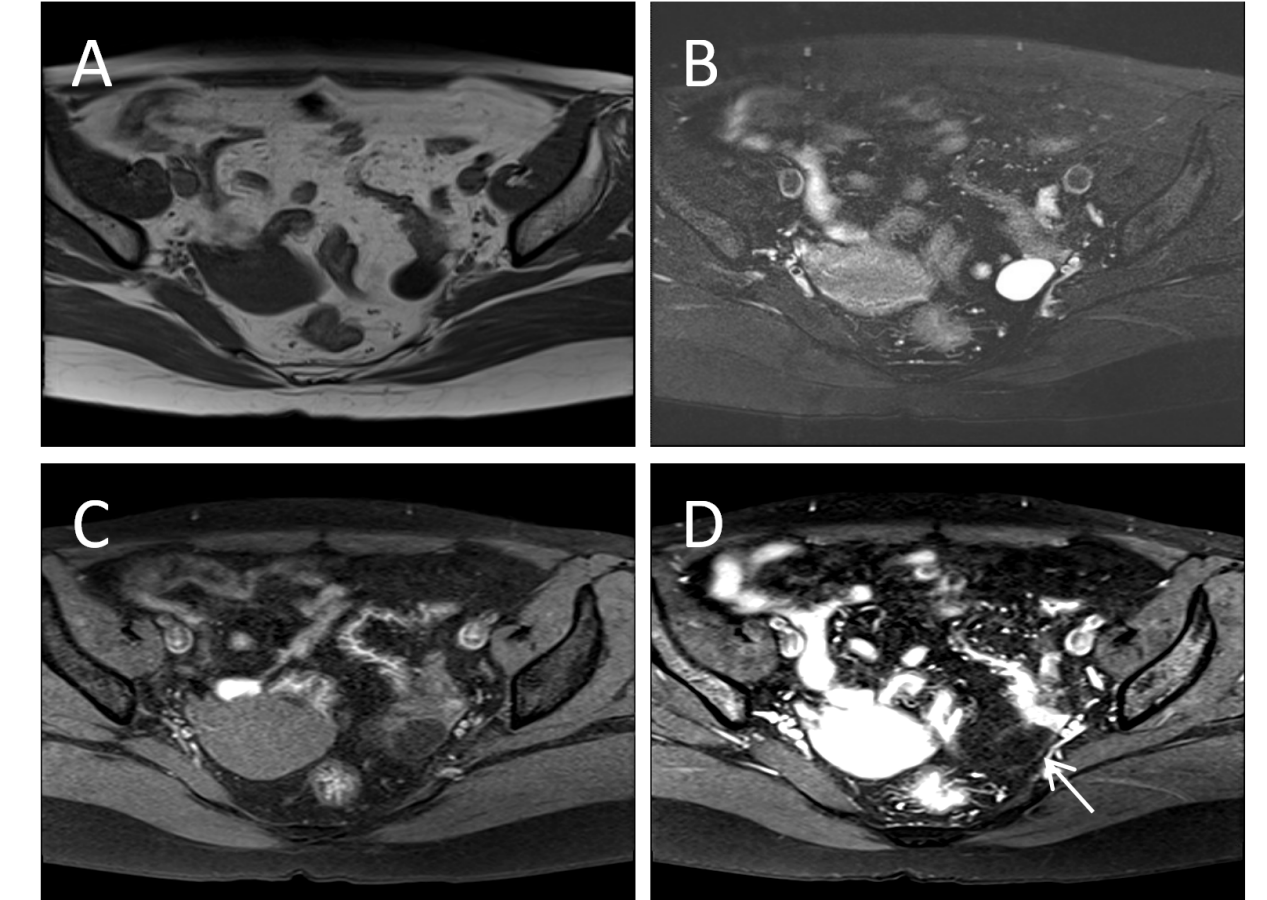


Supplementary Figure 1

A 35-year-old patient with a left ovarian serous cystadenoma was presented with a cystic mass in the left pelvic cavity on T1WI (A) and T2WI-FS (B), showing homogeneous signal without solid components. Enhanced T1WI-FS (D) revealed mild enhancement of the cyst wall (arrow), without solid nodules or protrusions. Determination result: cystic.


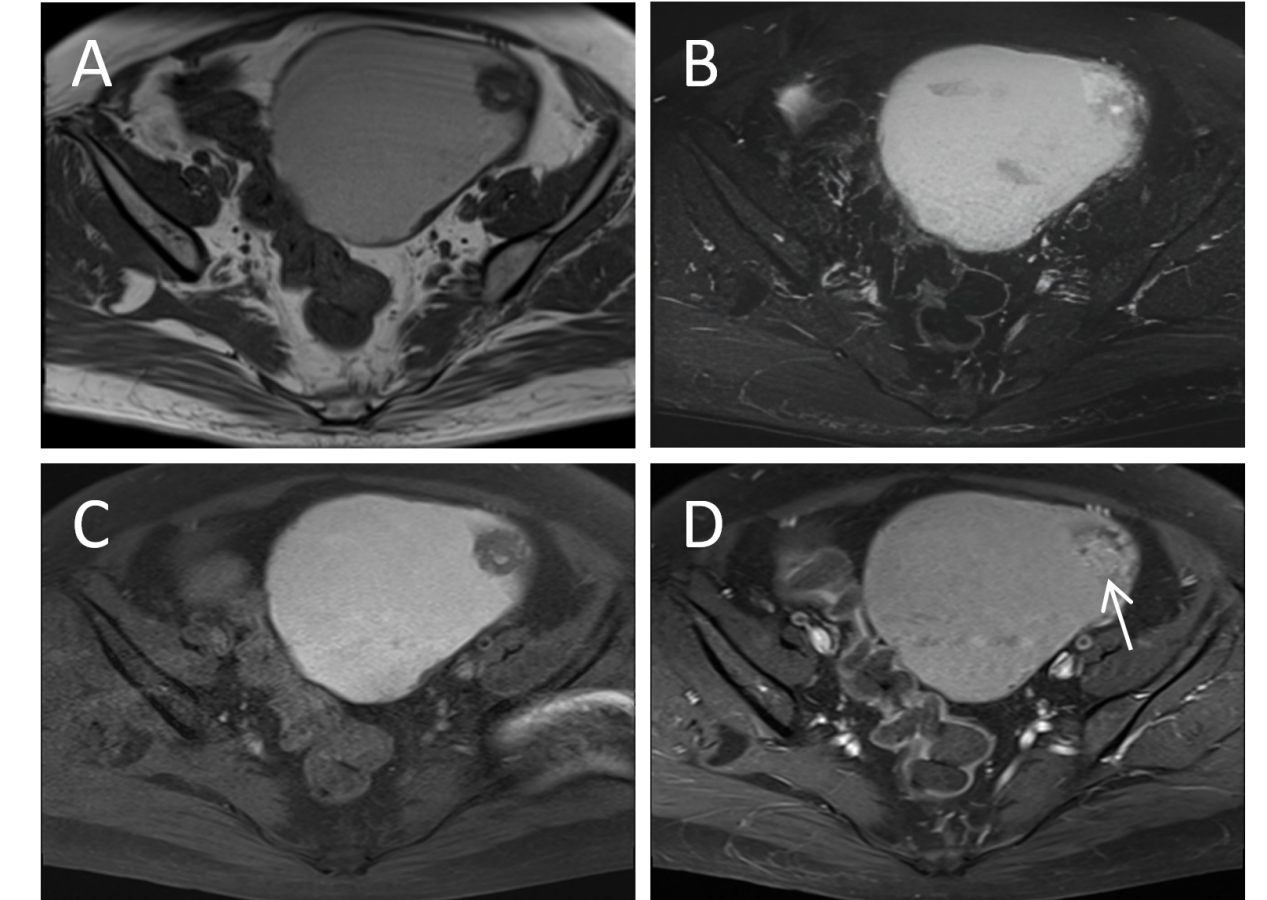


Supplementary Figure 2

A 47-year-old patient with a mature cystic teratoma in the left ovary was diagnosed as cystic-solid tumor on T1WI (A) and T2WI-FS (B), showing solid components within the mass. Enhanced T1WI-FS (D) revealed increased signal intensity in the solid components (arrow). Determination result: cystic-solid.


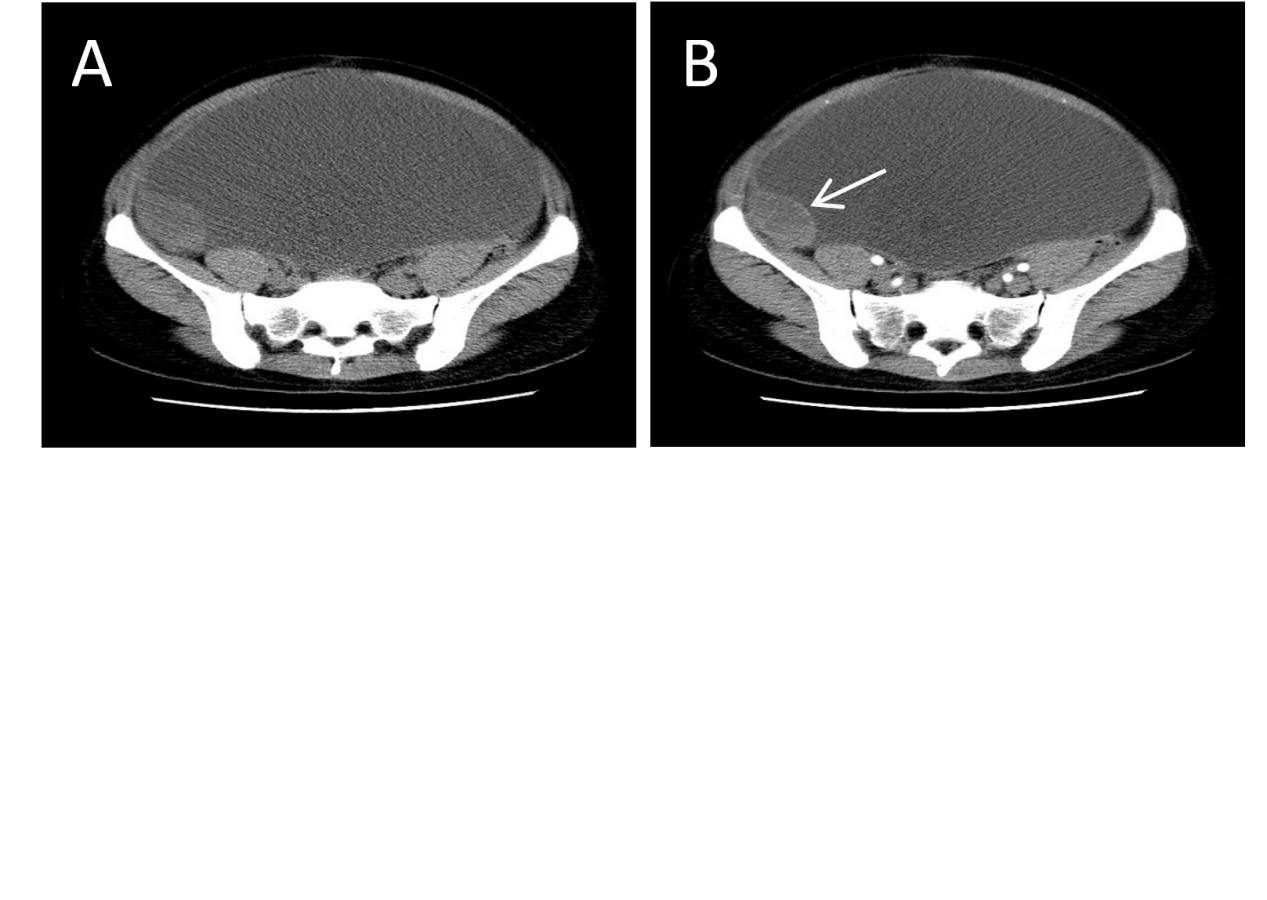


Supplementary Figure 3

A 51-year-old patient with a right ovarian mucinous cystadenoma was presented with a pelvic mass on non-contrast CT (A), and contrast-enhanced CT (B) showing well-demarcated solid and cystic components without infiltration (arrow). Determination result: cystic-solid interface clear.


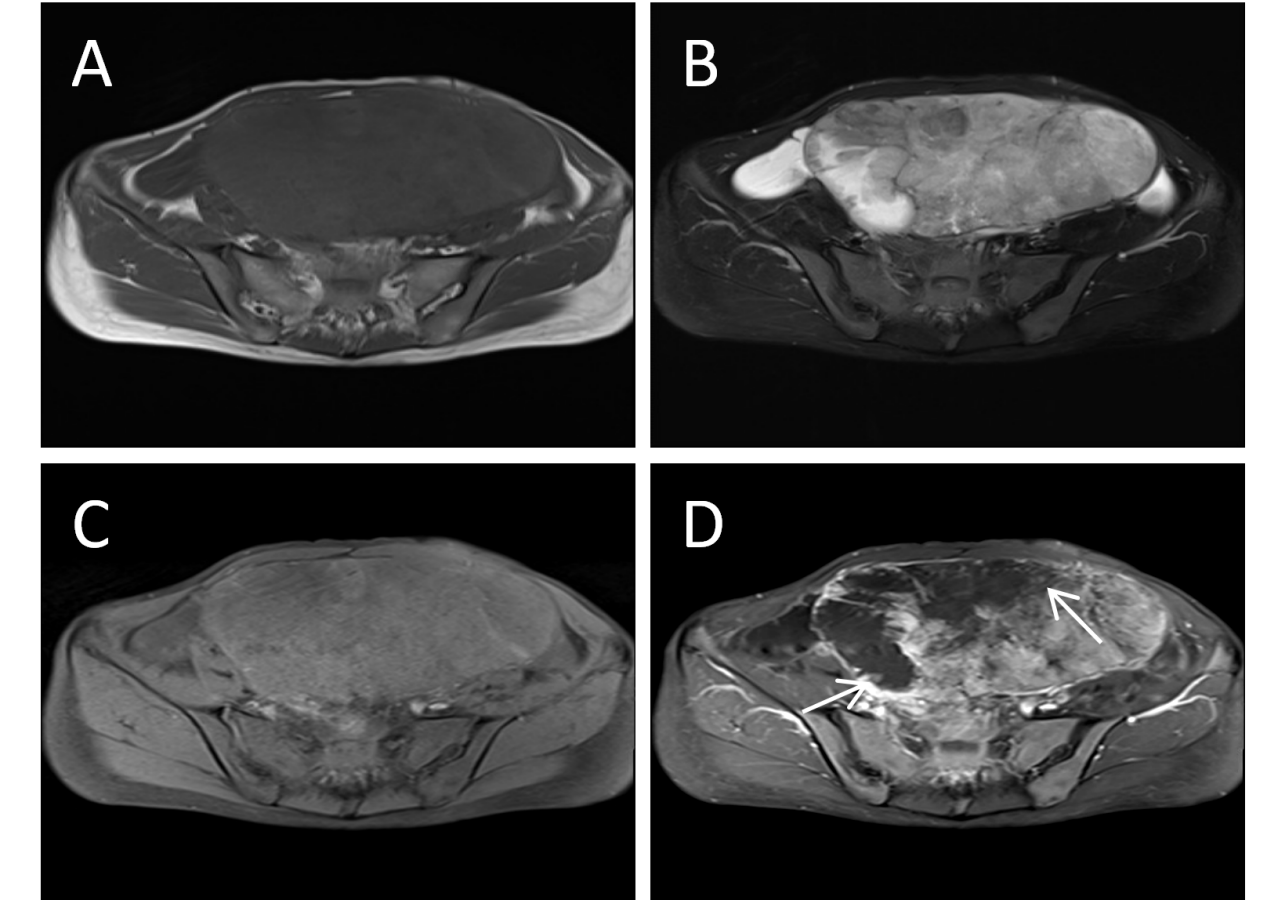


Supplementary Figure 4

A 44-year-old patient with left ovarian clear cell carcinoma was presented with a pelvis mass on T1WI (A) and T2WI-FS (B). Enhanced T1WI-FS (D) showed infiltration of solid components into cystic components with rough and blurred borders (arrow). Determination result: cystic-solid interface unclear.


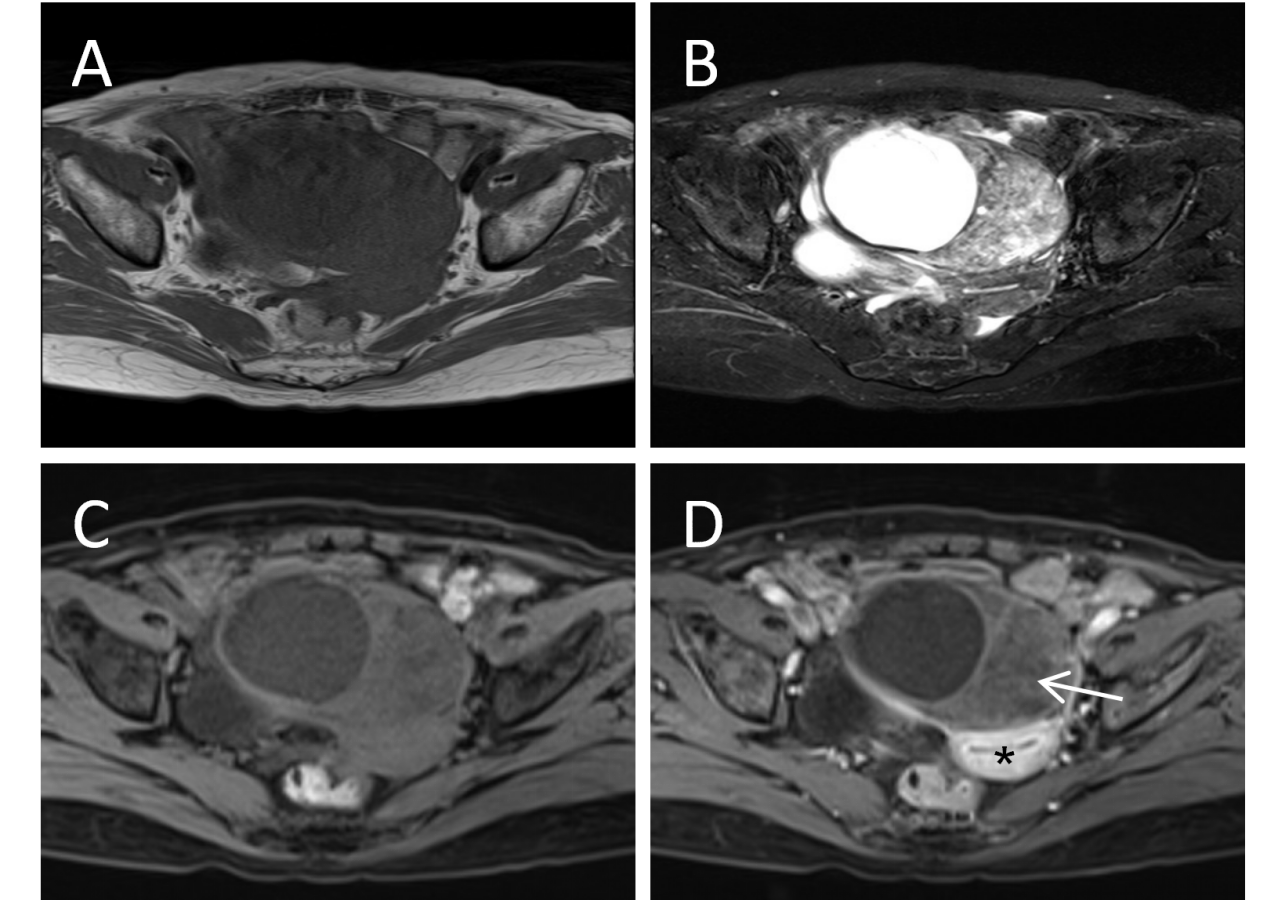


Supplementary Figure 5

A 52-year-old patient with a left ovarian thecoma-fibroma tumor. T1WI (A) and T2WI-FS (B) showed a solid unilocular mass in the pelvis, with mild enhancement (arrow) on enhanced T1WI-FS (D). The signal intensity was significantly lower than that of the myometrium (_*_). Determination result: mild enhancement.


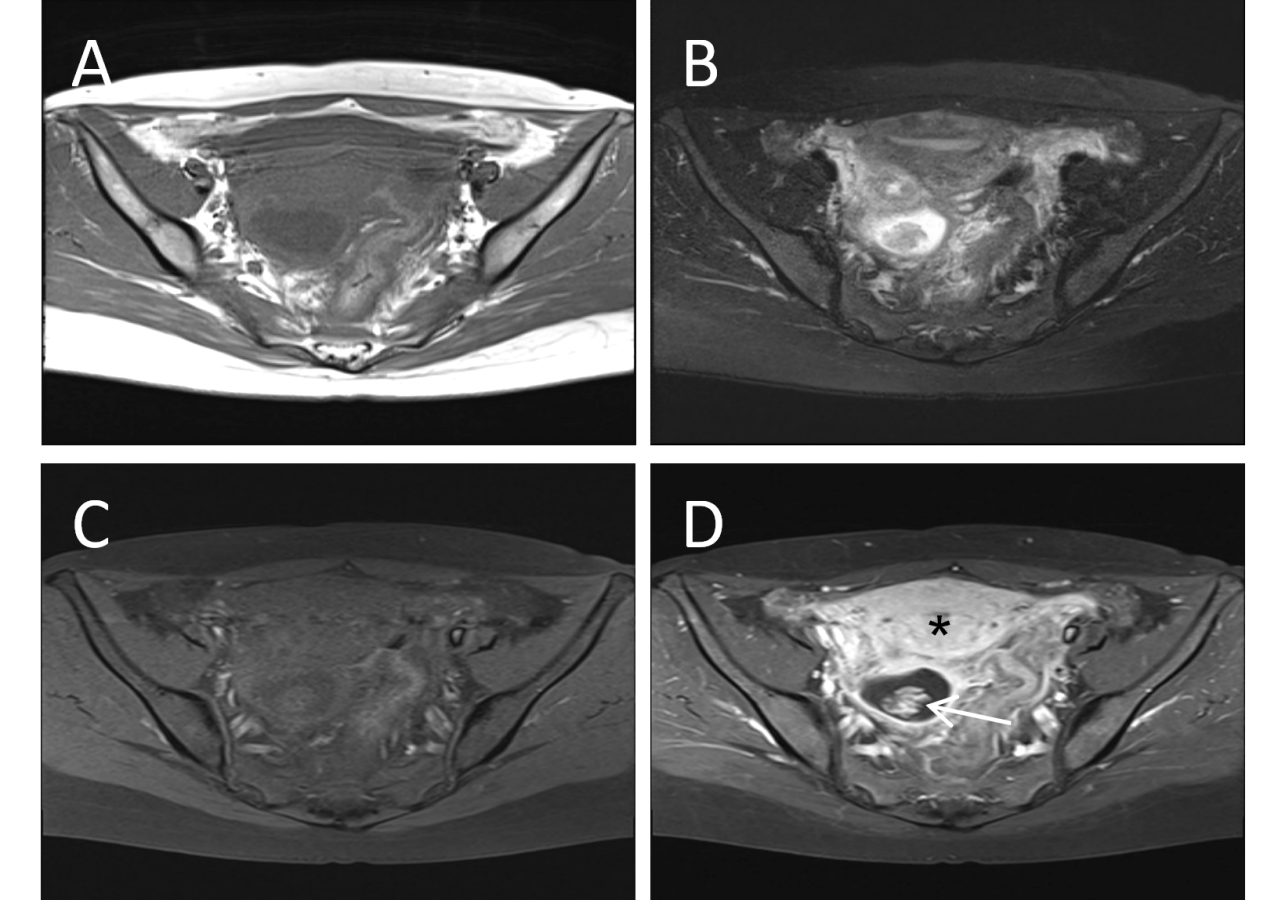


Supplementary Figure 6

A 65-year-old patient with right ovarian serous cystadenocarcinoma was diagnosed. T1WI (A) and T2WI-FS (B) revealed a pelvic cystic-solid mass, while enhanced T1WI-FS (D) showed solid component (arrow) with signal intensity slightly higher than the myometrium (_*_). Determination result: moderate enhancement.


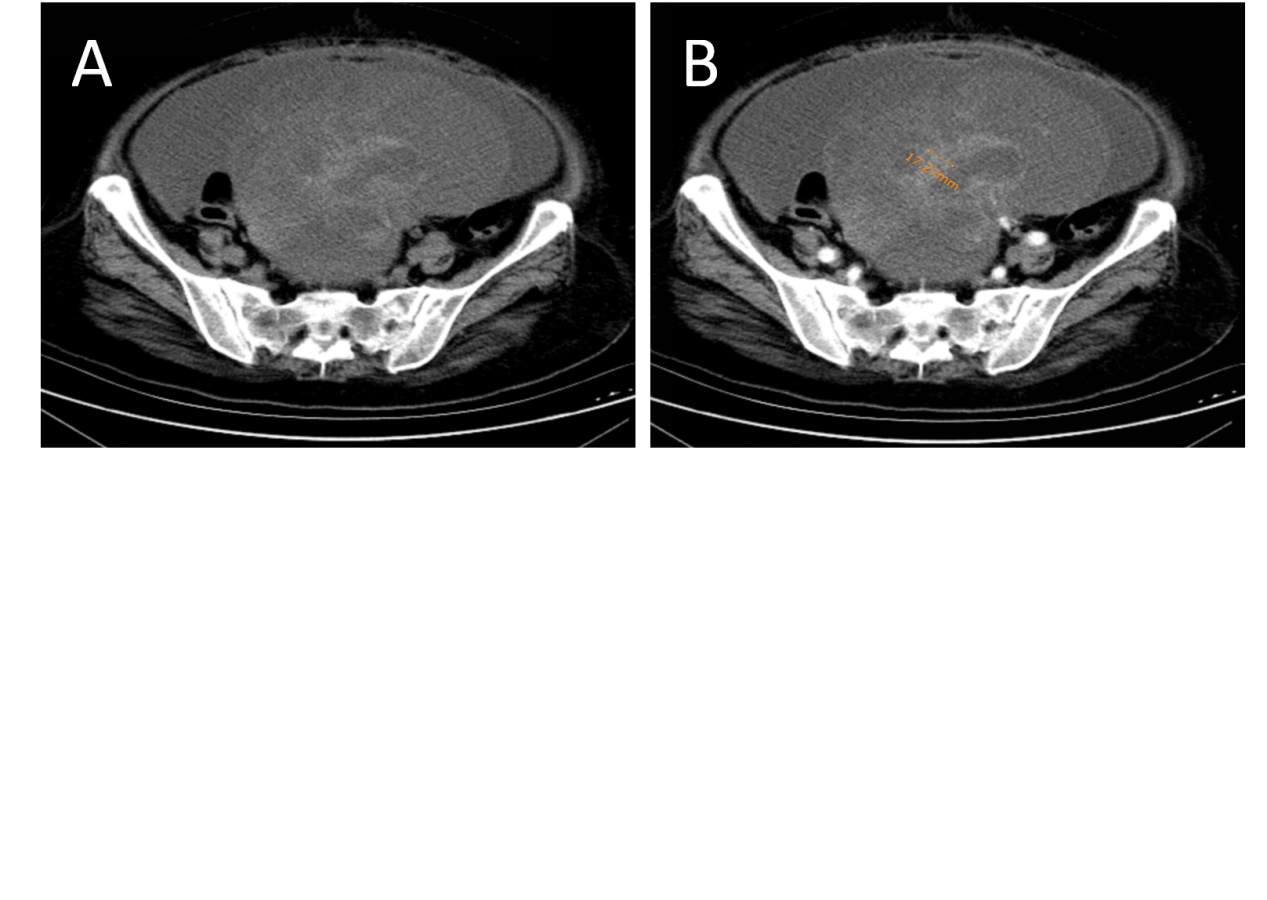


Supplementary Figure 7

A 74-year-old patient with right ovarian mucinous adenocarcinoma. Non-contrast CT (A) showed a pelvic multilocular cystic mass with significantly thickened septum. The thickest septum was measured on contrast-enhanced CT (B) and was about 17.27mm. Determination result: septum thickness ≥4 mm.

Supplementary Table 1 Univariate analysis of the full variables

| Variables | B | SE | *P* value | 95% CI |
| --- | --- | --- | --- | --- |
| CA125 elevated | 2.763 | 0.618 | **＜0.001** | 1.552-3.974 |
| CA15-3 elevated | 0.892 | 0.521 | **0.001** | 0.131-1.915 |
| Maximum diameter of solid portion (mm) | 0.215 | 0.043 | **＜0.001** | 0.131-0.299 |
| Maximum diameter ratio of solid portion | 3.826 | 0.915 | **＜0.001** | 2.031-5.621 |
| Location (bilateral) | 1.207 | 0.418 | **0.003** | 0.388-2.026 |
| Texture (cystic-solid) | 4.982 | 1.763 | **＜0.001** | 1.527-8.437 |
| Shape (irregular) | 1.873 | 0.526 | **＜0.001** | 0.839-2.907 |
| Margin (unsmooth) | 0.765 | 0.632 | **＜0.001** | 0.278-2.008 |
| Cystic-solid interface (unclear) | 5.126 | 1.689 | **＜0.001** | 1.811-8.441 |
| Septum thickness (≥4mm) | 1.024 | 0.401 | **＜0.001** | 0.237-1.811 |
| Enhancement degree (moderate or prominent) | 1.568 | 0.482 | **＜0.001** | 0.623-2.513 |
| Ascites (moderate/massive) | 0.937 | 0.542 | **＜0.001** | 0.126-1.998 |
| Lymph node (≥1cm) | 1.724 | 0.571 | **＜0.001** | 0.605-2.843 |

Bold values indicate statistical significance, with *p* < 0.05.

Supplementary Table 2 Summary table of the scoring system for clinical use in BCOTs

|  | Histological subtypes | Number | Scoring range | Average score |
| --- | --- | --- | --- | --- |
| BCOTs | Serous cystadenoma | 67 | 0 - 9 | 2.03 |
|  | Mucinous cystadenoma | 6 | 0 - 7 | 3.17 |
|  | Thecoma-fibroma tumor | 3 | 0 - 9 | 5.67 |
|  | Clear cell tumor | 2 | 5 - 7 | 6.00 |
|  | Mature cystic teratoma | 20 | 5 - 9 | 5.85 |
|  | Struma ovarii | 1 | 6 - 6 | 6.00 |
|  | Brenner tumor | 1 | 5 - 5 | 5.00 |

Supplementary Table 3 Summary table of the scoring system for clinical use in MCOTs

|  | Histological subtypes | Number | Scoring range | Average score |
| --- | --- | --- | --- | --- |
| MCOTs | Serous cystadenocarcinoma | 58 | 5 - 10 | 8.78 |
|  | Mucinous cystadenocarcinoma | 19 | 2 - 10 | 7.74 |
|  | Clear cell carcinoma | 10 | 5 - 10 | 7.50 |
|  | Yolk sac tumor | 5 | 9 - 10 | 9.20 |
|  | Endometrioid carcinoma | 2 | 9 - 10 | 9.50 |
|  | Granulosa cell tumor | 2 | 9 - 10 | 9.50 |

Supplementary Table 4 The baseline characteristics and diagnostic performance of the subgroups.

| Index | MRI-only(n=141) | CT-only(n=55) | *P* value |
| --- | --- | --- | --- |
| Tumor nature (benign/malignant) | 72/69 | 28/27 | 0.913 |
| Proportion of positive predictors in the scoring model (malignant tumors) |  |  |  |
| Cystic-solid components | 68/69（98.6%） | 27/27（100%） | 0.987 |
| Septum thickness (≥4mm) | 51/69（73.9%） | 20/27（74.1%） | 0.215 |
| Moderate or prominent  enhancement degree | 65/69（94.2%） | 25/27（92.6%） | 0.883 |
| CA125 elevated (malignant tumor) | 55/69（79.7%） | 21/27（77.8%） | 0.821 |
| AUC (95%CI) | 0.948（0.912-0.984） | 0.923（0.865-0.981） | 0.502 |
| Sensitivity | 85.7%（75.1%-93.2%） | 83.3%（65.3%-94.4%） | 0.778 |
| Specificity | 90.2%（80.3%-96.4%） | 88.9%（71.9%-97.7%） | 0.835 |
| PPV | 89.7% | 87.5% | - |
| NPV | 86.3% | 85.2% | - |

Bold values indicate statistical significance, with *p* < 0.05.
